# Phase I pharmacokinetic study of an oral, small-molecule MEK inhibitor tunlametinib in patients with advanced NRAS mutant melanoma

**DOI:** 10.3389/fphar.2022.1039416

**Published:** 2022-11-01

**Authors:** Qian Zhao, Teng Wang, Huanhuan Wang, Cheng Cui, Wen Zhong, Diyi Fu, Wanlin Xi, Lu Si, Jun Guo, Ying Cheng, Hongqi Tian, Pei Hu

**Affiliations:** ^1^ Clinical Pharmacology Research Center, Peking Union Medical College Hospital, Chinese Academy of Medical Sciences & Peking Union Medical College, State Key Laboratory of Complex Severe and Rare Diseases, NMPA Key Laboratory for Clinical Research and Evaluation of Drug, Beijing Key Laboratory of Clinical PK and PD Investigation for Innovative Drugs, Beijing, China; ^2^ Key Laboratory of Carcinogenesis and Translational Research (Ministry of Education), Department of Renal Cancer and Melanoma, Peking University Cancer Hospital and Research Institute, Beijing, China; ^3^ Shanghai KeChow Pharma, Inc., Shanghai, China

**Keywords:** melanoma, NRAS mutation, mek inhibitor, pharmacokinetics, tunlametinib

## Abstract

**Background:** Malignant melanoma is an aggressive disease. Tunlametinib (HL-085) is a potent, selective, and orally bioavailable MEK1/2 inhibitor. The objective of this study was to determine the pharmacokinetics (PK) of tunlametinib and its main metabolite M8 in patients with *NRAS*-mutant melanoma following a single dose and multiple doses in a phase I safety and PK study.

**Methods:** A multiple-center phase I study was performed in patients with melanoma including dose-escalation phase and dose-expansion phase. PK following a single oral dose and multiple doses of 0.5–18 mg twice daily was assessed.

**Results:** A total of 30 participants were included in the dose escalation phase and then 11 patients were included in the dose-expansion phase (12 mg twice daily). Tunlametinib plasma concentration rapidly increased after dosing, with a T_max_ of 0.5–1 h. Mean elimination half-life (t_1/2_) was dose-independent and had a range from 21.84 to 34.41 h. Mean apparent clearance (CL/F) and distribution volume (V/F) were 28.44–51.93 L/h and 1199.36–2009.26 L, respectively. The average accumulation ratios of AUC and C_max_ after the multiple administration of tunlametinib were 1.64–2.73 and 0.82–2.49, respectively. Tunlametinib was rapidly transformed into the main metabolite M8 and M8 reached the peak concentration about 1 h after administration. Mean t_1/2_ of M8 was 6.1–33.54 h. The body exposure of M8 in plasma was 36%–67% of that of tunlametinib. There were general dose-proportional increases in maximum concentration (C_max_) and area under the curve (AUC) of tunlametinib and M8 both in the single dose phase and in the multiple doses phase.

**Conclusion:** Tunlametinib was absorbed rapidly and eliminated at a medium speed after drug withdrawal. Pharmacokinetic body exposure increased in general dose-proportional manner from 0.5 mg up to 18 mg. Slight accumulation was found after multiple oral doses. The pharmacokinetics of tunlametinib and its metabolite suggest that twice daily dosing is appropriate for tunlametinib.

## Background

Melanoma is a malignance of melanocytes and an aggressive disease, which was recognized as the most dangerous type of skin cancer (Schadendorf et al., 2018). The five world regions with the greatest melanoma incidence and mortality rates were Australia, North America, Eastern Europe, and Western Europe and Central Europe (Karimkhani et al., 2017). The incidence of melanoma is keeping a worldwide increase. Incidence in Europe is about 25 cases per 100,000 population, while in Australia it reaches a rate of 60 new cases per 100,000 (Podlipnik et al., 2020; Conforti and Zalaudek, 2021). Total melanoma incidence was higher in male than female in US individuals (limited to white race), Canada, Australia, and New Zealand. Meanwhile, and male had higher rates of melanoma of the head and neck and trunk than female (Olsen et al., 2020). The age-standardized incidence rate of melanoma has increased from 0.4 per 100,000 in 1990 to 0.9 per 100,000 in 2017 in China (Wu et al., 2020). The median survival was about 1 year for advanced metastatic melanoma (Tsao et al., 2004). For 40 years, few effective systemic treatments to melanoma are available. The standard-of-care treatments included dacarbazine chemotherapy and immunotherapy with the cytokine IL-2/PD-1/PD-L1/CTLA-4. In the past 10 years, new targeted therapy has changed the treatment option for patients with metastatic melanoma (Luke et al., 2017; Schadendorf et al., 2018; Davis et al., 2019; Jenkins and Fisher, 2021).

Activating mutations in the MAPK signaling cascade was an important pathway that has the highest oncogenic and therapeutic relevance for melanoma (Carlino et al., 2015). Common mutations in the MAPK pathway include *BRAF*, *NRAS*, *HRAS*, *KRAS* and *NF1* (Schadendorf et al., 2015; Randic et al., 2021). BRAF signaling is dependent on downstream activation of MEK1/2 (Solit et al., 2006). Thus, several MEK inhibitors have been developed for melanoma treatment alone or in combination (Del Vecchio et al., 2015; Robert et al., 2015; Ascierto et al., 2016; Dummer et al., 2017). MEK inhibitors trametinib, cobimetinib and binimetinib have been approved for BRAF V600 melanoma by the Food and Drug Administration (Kakadia et al., 2018). However, no MEK inhibitor was approved for melanoma patients with *NRAS* mutation worldwide.

Tunlametinib (also known as HL-085) is a selective inhibitor of MEK1 and MEK2 with a half-maximum inhibitory concentration (IC_50_) of 1.9–10 nmol/L (Cheng and Tian, 2017). Tunlametinib inhibited proliferation of RAS/RAF-mutated cell lines at nanomoles concentrations (unpublished investigator brochure, Shanghai KeChow Pharma.). Pharmacokinetic profiling results indicated a mean effective half life (t_1/2_) of 3.55–4.62 and 3.99–9.37 h in rats and beagle dogs after single oral dosing of tunlametinib. CYP2C9 was the main metabolic enzyme of tunlametinib. The main metabolite M8 is inactive. >60% of ^14^C-tunlametinib were excreted from feces in rats (unpublished investigator brochure, Shanghai KeChow Pharma.). Tunlametinib may be a potential treatment option for *NRAS*-mutant melanoma.

Tunlametinib was recently assessed in first-in-human trial: a single ascending-dose and a multiple ascending-dose phase I study in patients with advanced NRAS-mutated melanoma patients, which evaluated the safety, tolerability, and pharmacokinetics of tunlametinib in melanoma patients. Presented here are the pharmacokinetic data from this phase I study.

## Methods

### Study design and patients

This study was an open, single-arm, dose-escalation/dose-expansion phase I trial including two parts: a dose-escalation phase (Part 1) and an expansion phase (Part 2). The Part 1 dose-escalation phase adopted a standard 3 + 3 design to evaluate the pharmacokinetic characters of tunlametinib, and to identify the dose-limiting toxicity (DLT), maximum-tolerated dose (MTD), recommended Phase II dose (RP2D). According to a 3 + 3 design, at least three patients were treated at each dose level. In the Part 2 dose-expansion phase, patients were administered at the RP2D to further evaluate the tolerability, safety, and efficacy of tunlametinib. The design of this study was presented in the Supplementary Figure S1.

Data for pharmacokinetics are reported here while other data will be reported separately. The study protocol was approved by local Ethics Review Committee and registered at clinicaltrials.gov (NCT03973151). All patients provided written informed consent. Studies were conducted in accordance with Declaration of Helsinki, Good Clinical Practice and applicable laws and regulations.

Eligible patients were aged 18–70 years with histologically or cytologically confirmed unresectable stage III or IV melanoma harboring *NRAS* mutations. Tumor biopsy was adequate for genetic testing of NRAS mutations. Patients were also required an Eastern Cooperative Oncology Group (ECOG) performance score of one or less; with measurable lesions per Response Evaluation Criteria in Solid Tumors (RECIST) version 1.1; life expectancy of >3 months and adequate hematologic, renal, and hepatic function.

Patients were excluded if they had active central nervous system disease except for patients with stable brain disease for ≥3 months following stereotactic brain radiotherapy or surgery; inability to swallow or any small intestinal resection that would preclude adequate absorption of the study drug; uncontrolled concomitant or infectious diseases; history of retinal disease; prior treatment with a specific MEK inhibitor; or known allergy to the study drug or its analogs. Strong inducers or inhibitors of CYP isozyme had to be discontinued ≥1 week before study treatment.

### Procedures

This dose-escalation study experienced 10 dose-levels, including 0.5, 1, 2, 3, 4, 6, 9, 12, 15, and 18 mg, and all the patients were administered tunlametinib capsule twice daily except in the PK lead-in period. In dose-escalation phase, a 7-day pharmacokinetic lead-in period was designed for each patient before entering treatment cycles. During the PK lead-in period, all the patients were administered only one dose. In dose-expansion phase, patients were given the recommended phase II dose (12 mg BID) without lead-in period.

To assess the pharmacokinetic profile of tunlametinib and its main metabolite M8 (inactive metabolite), serial venous blood samples were collected after overnight fasting on day -7 (single dose, lead-in period) and day 28 (multiple doses).

In the lead-in period, blood samples were drawn pre-dose (0 h) and at 1, 2, 3, 4, 8, 12, 24, 48, 72, 96, 120, and 144 h after tunlametinib administration in the dose cohorts of 0.5 and 1 mg; This was optimized to pre-dose and at 0.25, 0.5, 1, 2, 4, 8, 12, 24, 48, 72, 96, 120, and 144 h after dosing for the following dose cohorts. On day 28, total seven time points of blood were collected pre-dose and 1, 2, 3, 4, 8, and 12 h post dose in the 0.5 mg cohort; and this was changed into total 8 time points including pre-dose and 0.25, 0.5, 1, 2, 3, 4, 8, and 12 h post dose in the other doses cohorts.

In the dose-expansion phase, total 8 time points of blood were taken at pre-dose and at 0.25, 0.5, 1, 2, 4, 8, and 12 h post dose on both day 1 and day 28 of cycle 1.

Sparse sampling was also collected on days 8, 15, and 22 of cycle one at pre-dose in both dose-escalation and dose-expansion phases.

### Sample processing and bioanalysis methods

Blood samples were collected into vacutainer tubes with EDTA-K_2_ anticoagulation. Immediately after collection, the blood-containing tubes were centrifuged at 1500 g at 4°C for 10 min. All the plasma samples were stored in a freezer at −80°C until subsequent bioanalytical analysis.

Plasma samples were assayed for tunlametinib and M8 concentrations at Peking Union Medical College Hospital using a validated ultra-performance liquid chromatography-tandem mass spectrometry method. Samples were prepared by using a solid phase extraction method. The quantification range for tunlametinib and M8 in plasma were 0.1–100 ng ml^−1^ [ 0.3, 8, and 80 ng ml^−1^ for quality controls (QCs)]. Stable isotope labeled tunlametinib (D_4_-tunlametinib) and M8 (D_3_-M8) were used as an internal standard, respectively. The lower limit of quantitation of tunlametinib and M8 in plasma were 0.1 ng·mL^−1^. The accuracy of the QC samples used during sample analysis ranged from -1.5% to 4.9% [relative standard deviation (RSD)% ≤ 13.2%] for tunlametinib and from -2.1% to 3.3% (RSD% ≤ 16.1%) for M8. All samples were analyzed within established storage stability periods.

### Study outcomes

The parameters assessed during the study were maximum observed plasma concentration (C_max_), time to C_max_ (T_max_), area under the plasma concentration-time curve from the time of dosing extrapolated to infinity (AUC_inf_), area under the concentration-time curve during a dosing interval at steady-state (AUC_τ_), terminal elimination half-life (t_1/2_), apparent clearance (CL/F), apparent volume of distribution during the terminal elimination phase (V_z_/F), accumulation ratio calculated from AUC_τ (day28)_ and AUC_0−12 h (PK lead-in period or day 1)_.

### Statistical analysis

The pharmacokinetic analysis set (PKAS) were used for the analysis of all pharmacokinetic data. The PKAS included all participants who took at least one dose of tunlametinib and had at least one collecting sample point parameter.

Plasma concentration-time data were analyzed and the PK parameters were calculated *via* non-compartmental analysis method using WinNonlin (version 8.3, Pharsight Corporation, United States). C_max_ and T_max_ were determined directly from experimental observations. AUC_τ_ was calculated using the linear trapezoidal method (linear up and log down). The first order rate constant (λ_z_) of decline in tunlametinib and M8 plasma concentrations in the terminal phase of the plasma concentration-time curve was estimated using linear regression. The t_1/2_ was estimated from ln2/λ_z_. AUC_inf_ was calculated using the following equation: AUC_0-t_ + AUC_Ex_, where AUC_0-t_ was the area under the concentration-time curve from time zero (pre-dose) to the time of the last quantifiable concentration, and AUC_Ex_ was the observed concentration at last sampling time divided by λ_z_. CL/F was calculated as the dose divided by AUC_inf_, and V_z_/F was estimated by dividing the apparent CL by λ_z_. The accumulation ratio for tunlametinib and M8 at steady state was determined by dividing the AUC_τ_ (or C_max_) on day 28 by the AUC_0–12 h_ (or C_max_) on PK lead-in period. PK parameters of tunlametinib and M8 were summarized using descriptive statistics, including mean, coefficient of variation, median, minimum, maximum, and geometric mean, where applicable.

Dose-exposure relationship after single- and multiple-dose administration of tunlametinib capsule was evaluated. Dose proportionality using AUC_inf_ and C_max_ over the administered dose range was determined by using a power model: log (parameter) = α + β log(dose) where α was the intercept and β was the slope. β = 1 + Lnθ/Lnr, where r was the ratio of high dose divided by low dose (for 0.5–18 mg dose range, r = 36), θ was the acceptance limit (lower limit θ_L_ = 0.80, upper limit θ_H_ = 1.25). θ_L_ < r^β−1^< θ_H_, dose proportionality was assessed based on whether the 90% CI constructed for the estimate of r^β−1^ was within the acceptance interval (0.80–1.25), that is to say, whether the 90% CI of β was within the acceptance interval (0.938.1.062).

## Results

### Demographics

A total of 30 participants were included in the dose escalation phase and then 11 patients were included in the dose-expansion phase (12 mg twice daily). The demographic characteristics of 41 patients are summarized in Table 1. The range of age was from 34 to 69 years and males and females accounted for 51.2% and 48.8%, respectively. Overall median body mass index (BMI) was in the 18.43–36.00 kg/m^2^ range.

**TABLE 1:** Demographics characteristics of participants treated with tunlametinib.

Characteristics	0.5 mg (*n* = 3)	1 mg (*n* = 3)	2 mg (*n* = 3)	3 mg (*n* = 3)	4 mg (*n* = 3)	6 mg (*n* = 3)	9 mg (*n* = 3)	12 mg (*n* = 14)*	15 mg (*n* = 3)	18 mg (*n* = 3)	Total (*n* = 41)
Age (years)	55.0 (36–63)	54.0 (43–62)	46.0 (43–52)	50.0 (39–55)	62.0 (45–69)	58.0 (44–68)	58.0 (56–59)	56.5 (41–67)	57.0 (34–58)	59.0 (56–63)	56.0 (34–69)
Sex-no. (%)											
Female	2 (66.7%)	1 (33.3%)	1 (33.3%)	1 (33.3%)	2 (66.7%)	1 (33.3%)	1 (33.3%)	7 (500%)	3 (100%)	1 (33.3%)	20 (48.8%)
Male	1 (33.3%)	2 (66.7%)	2 (66.7%)	2 (66.7%)	1 (33.3%)	2 (66.7%)	2 (66.7%)	7 (50.0%)	0	2 (66.7%)	21 (51.2%)
Height (cm)	160.00 (154.0–172.0)	166.00 (148.0–167.0)	165.00 (160.0–172.0)	173.00 (154.0–180.0)	161.00 (150.0–170.0)	167.00 (159.0–175.0)	165.00 (158.0–171.0)	164.00 (158.0–175.0)	160.00 (153.0–163.0)	158.00 (157.0–159.0)	163.00 (148.0–180.0)
Weight (kg)	56.00 (48.0–72.0)	69.00 (62.0–85.0)	72.00 (62.0–84.0)	67.00 (55.0–75.0)	61.00 (60.0–78.0)	68.00 (67.4–87.0)	56.00 (49.0–88.0)	65.75 (46.0–98.0)	64.00 (54.0–75.0)	66.30 (59.0–71.5)	67.00 (46.0–98.0)
BMI (kg/m^2^)	21.88 (20.24–24.34)	28.31 (25.04–30.48)	24.34 (22.77–32.81)	23.15 (22.39–23.19)	26.67 (23.53–26.99)	26.90 (24.17–28.41)	20.57 (19.63–30.09)	23.56 (18.43–36.00)	25.00 (20.32–32.04)	26.90 (23.34–28.64)	24.17 (18.43–36.00)

Data was presented as median and range (min, max) unless otherwise specific indicated.

BMI: body mass index.

*12 mg cohort include 3 patients in dose-escalation phase and 11 patients in dose-expansion phase.

### Single-dose pharmacokinetics

**FIGURE 1:**
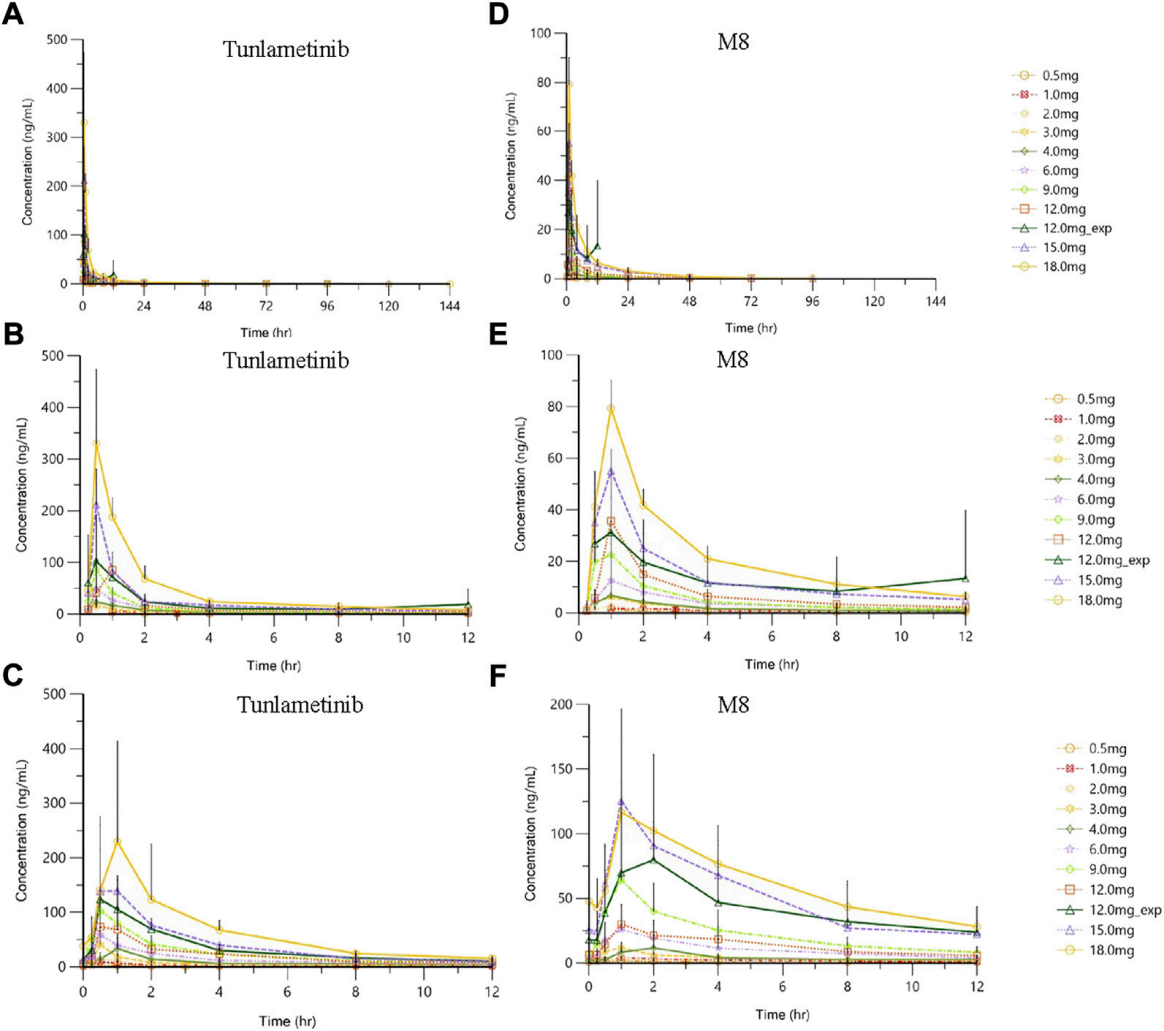
| Plasma concentration (mean ± SD)-time profiles of tunlametinib and M8 after orally administrated with 0.5–18 mg (*n* = 41) tunlametinib capsules. **(A)** single dose (0–144 h, tunlametinib); **(B)** single dose (0–12 h, tunlametinib); **(C)** multiple dose (tunlametinib); **(D)** single dose (0–144 h, M8); **(E)** single dose (0–12 h, M8); **(F)** multiple dose (M8).

Tunlametinib and M8 plasma concentration increased and reached to the peak concentration fast after tunlametinib administration, and then declined slowly. The concentration-time curve of tunlametinib and M8 was shown in Figure 1. Pharmacokinetics parameters of tunlametinib and M8 were shown in the Table 2.

**TABLE 2:** Summary of pharmacokinetic parameters of tunlametinib and M8.

Dose-escalation phase													Expansion phase
	Parameter	Unit	0.5 mg (*n* = 3)	1.0 mg (*n* = 3)	2.0 mg (*n* = 3)	3.0 mg (*n* = 3)	4.0 mg (*n* = 3)	6.0 mg (*n* = 3)	9.0 mg (*n* = 3)	12.0 mg (*n* = 3)	15.0 mg (*n* = 3)	18.0 mg (*n* = 3)	12.0 mg (*n* = 11)
HL-085													
Single dose	AUC_0–12h_	h*ng/mL	5.69 ± 0.93	17.25 ± 7.03	23.34 ± 6.85	37.20 ± 10.83	53.91 ± 12.05	84.61 ± 40.93	114.33 ± 43.22	147.6 ± 17.70	273.89 ± 21.81	488.89 ± 50.59	228.96 ± 77.81
	AUC_inf_	h*ng/mL	/	42.76 ± 33.97	43.62 ± 9.57	73.49 ± 30.65	89.5 ± 25.29	144.48 ± 57.73	183.72 ± 33.90	232.69 ± 23.17	398.85 ± 34.57	635.74 ± 53.55	/
	AUC__%Extrap_	%	25.74 ± 4.06	17.05 ± 4.93	10.94 ± 3.66	6.86 ± 3.25	6.03 ± 1.61	6.18 ± 2.41	3.10 ± 0.97	2.61 ± 0.96	1.33 ± 0.36	0.83 ± 0.27	26.83 ± 27.56
	C_max_	ng/mL	2.24 ± 0.99	6.98 ± 4.03	15.70 ± 2.39	19.60 ± 6.28	27.60 ± 4.75	54.07 ± 31.98	95.27 ± 85.79	85.63 ± 24.15	211.67 ± 68.82	349.33 ± 111.13	142.83 ± 88.63
	C_last_	ng/mL	0.15 ± 0.06	0.15 ± 0.06	0.14 ± 0.03	0.12 ± 0.01	0.18 ± 0.07	0.19 ± 0.02	0.17 ± 0.03	0.16 ± 0.03	0.18 ± 0.05	0.14 ± 0.03	17.87 ± 27.52
	T_max_^a^	H	0.98 (0.98.1.00)	0.92 (0.92.1.00)	0.50 (0.50.1.00)	0.50 (0.48.0.98)	0.50 (0.50.1.00)	0.50 (0.48.2.00)	0.52 (0.50.1.00)	1.00 (0.98.1.00)	0.50 (0.50.0.50)	0.52 (0.50.0.97)	0.53 (0.23.11.65)
	MRT	H	6.48 ± 2.88	19.26 ± 10.86	14.42 ± 1.69	18.02 ± 5.64	13.66 ± 3.73	15.41 ± 2.92	14.25 ± 4.57	14.99 ± 5.03	12.85 ± 4.51	10.54 ± 2.44	/
	T_1/2_	H	/	34.41 ± 16.18	24.14 ± 3.15	28.2 ± 10.61	21.84 ± 5.66	30.79 ± 4.82	23.04 ± 5.52	26.18 ± 5.56	24.28 ± 10.33	29.13 ± 3.57	/
	CL/F	L/h	/	34.17 ± 27.15	47.32 ± 10.13	45.46 ± 17.04	47.43 ± 14.71	45.90 ± 16.74	50.10 ± 9.10	51.93 ± 5.47	37.81 ± 3.45	28.44 ± 2.28	/
	V_z_/F	L	/	1379.52 ± 550.08	1617.46 ± 141.04	1772.99 ± 788.61	1422.54 ± 200.07	2009.26 ± 651.60	1708.45 ± 655.99	1942.20 ± 311.42	1292.35 ± 470.57	1199.36 ± 214.11	/
Multiple dose	AUC_τ_	h*ng/mL	12.67 ± 3.02	31.56 ± 14.23	45.76 ± 12.31	76.42 ± 25.03	96.09 ± 47.77	163.95 ± 57.14	294.37 ± 114.84	266.55 ± 35.62	446.70 ± 73.76	906.33 ± 74.43	391.16 ± 97.85
	C_avg_	ng/mL	1.06 ± 0.25	2.63 ± 1.19	3.81 ± 1.03	6.37 ± 2.09	8.01 ± 3.98	13.66 ± 4.76	24.53 ± 9.57	22.21 ± 2.97	37.23 ± 6.15	75.53 ± 6.20	32.60 ± 8.15
	C_max_	ng/mL	2.52 ± 0.80	11.11 ± 9.57	14.50 ± 0.85	40.97 ± 25.44	33.23 ± 19.42	58.90 ± 37.74	109.40 ± 73.5	77.90 ± 46.49	186.33 ± 95.00	238.37 ± 168.72	140.57 ± 71.53
	C_min_	ng/mL	0.74 ± 0.24	1.51 ± 0.66	2.44 ± 0.06	2.87 ± 0.60	3.29 ± 3.11	4.40 ± 0.60	6.22 ± 1.25	5.95 ± 2.01	12.05 ± 2.95	14.23 ± 4.23	10.12 ± 4.93
	Fluctuation%	%	166.40 ± 21.09	332.28 ± 204.62	331.62 ± 113.12	583.46 ± 268.56	391.32 ± 114.01	391.19 ± 174.17	384.33 ± 140.35	448.51 ± 181.90	450.60 ± 172.16	418.85 ± 33.95	435.72 ± 216.88
	T_max_^a^	h	0.98 (0.97.0.98)	0.97 (0.52.1.02)	0.73 (0.50.0.97)	0.50 (0.48.0.50)	0.98 (0.95.1.00)	0.52 (0.50.1.00)	1.00 (0.50.1.02)	0.50 (0.50.2.08)	0.98 (0.53.1.00)	1.00 (0.98.4.02)	1.00 (0.48.2.03)
	R_1_	-	1.44 ± 1.09	1.47 ± 0.61	0.93 ± 0.25	2.49 ± 2.27	1.28 ± 0.93	1.23 ± 0.54	1.73 ± 1.63	0.99 ± 0.63	0.97 ± 0.67	0.82 ± 0.78	0.94 ± 0.29
	R_2_	-	2.32 ± 0.83	1.81 ± 0.18	2.42 ± 1.02	2.05 ± 0.36	1.83 ± 0.96	2.02 ± 0.46	2.73 ± 1.22	1.84 ± 0.55	1.64 ± 0.36	1.75 ± 0.09	1.59 ± 0.19
	R_3_	-	/	0.69 ± 0.20	1.22 ± 0.49	1.08 ± 0.30	1.14 ± 0.66	1.15 ± 0.14	1.61 ± 0.61	1.08 ± 0.13	1.12 ± 0.10	1.39 ± 0.03	/
**M8**													
Single dose	AUC_0–12h_	h*ng/mL	4.23 ± 1.22	9.76 ± 6.21	7.50 ± 2.22	22.28 ± 11.53	22.82 ± 20.69	43.64 ± 31.68	60.91 ± 46.38	83.93 ± 30.01	161.99 ± 27.4	245.33 ± 16.17	120.43 ± 90.82
	AUC_inf_	h*ng/mL	/	29.93 ± 27.99	9.76 ± 2.12	39.23 ± 25.87	33.00 ± 32.06	69.06 ± 46.60	89.19 ± 46.82	124.91 ± 28.5	261.27 ± 36.23	366.9 ± 48.25	/
	AUC__%Extrap_	%	26.39 ± 5.74	16.28 ± 6.21	17.65 ± 6.63	7.71 ± 5.84	8.70 ± 4.08	7.48 ± 7.13	5.49 ± 3.12	3.19 ± 2.24	1.40 ± 0.29	2.31 ± 1.02	19.55 ± 11.66
	C_max_	ng/mL	1.19 ± 0.33	2.03 ± 0.36	2.73 ± 0.81	6.84 ± 1.86	6.70 ± 5.06	15.24 ± 11.68	23.54 ± 23.87	35.63 ± 20.53	55.10 ± 8.31	79.37 ± 10.74	42.22 ± 25.87
	C_last_	ng/mL	0.13 ± 0.03	0.15 ± 0.08	0.20 ± 0.05	0.13 ± 0.03	0.17 ± 0.08	0.17 ± 0.03	0.21 ± 0.07	0.15 ± 0.08	0.20 ± 0.06	0.18 ± 0.02	12.36 ± 25.29
	T_max_^a^	h	1.00 (0.98.1.93)	0.92 (0.92.2.92)	0.97 (0.50.1.00)	0.98 (0.48.1.03)	0.97 (0.95.1.00)	1.00 (0.95.2.00)	0.98 (0.50.1.00)	1.00 (0.98.1.00)	1.00 (1.00.1.03)	1.00 (0.97.1.00)	1.00 (0.50.11.65)
	MRT	h	6.81 ± 2.45	13.85 ± 10.67	6.12 ± 2.01	12.14 ± 6.72	7.31 ± 2.49	10.55 ± 5.01	10.66 ± 3.52	12.04 ± 3.47	13.94 ± 2.32	12.70 ± 2.72	/
	T_1/2_	h	/	21.32 ± 13.74	6.10 ± 2.66	14.36 ± 5.22	9.89 ± 2.36	13.10 ± 3.87	12.79 ± 1.69	17.31 ± 2.74	15.75 ± 2.37	33.54 ± 11.84	/
	MPratio__AUC0–12h_	-	0.88 ± 00.36	0.67 ± 0.30	0.36 ± 0.01	0.65 ± 0.16	0.49 ± 0.43	0.55 ± 0.23	0.55 ± 0.22	0.65 ± 0.23	0.67 ± 0.06	0.57 ± 0.05	0.58 ± 0.31
	MPratio__AUCinf_	-	/	0.73 ± 0.16	0.25 ± 0.02	0.57 ± 0.15	0.42 ± 0.38	0.50 ± 0.23	0.53 ± 0.18	0.62 ± 0.21	0.75 ± 0.08	0.66 ± 0.07	/
	MPratio__Cmax_	-	0.76 ± 0.53	0.42 ± 0.25	0.20 ± 0.07	0.40 ± 0.02	0.28 ± 0.22	0.39 ± 0.26	0.26 ± 0.04	0.46 ± 0.18	0.31 ± 0.07	0.27 ± 0.06	0.44 ± 0.38
Multiple dose	AUC_τ_	h*ng/mL	8.90 ± 4.17	22.01 ± 10.25	16.45 ± 6.06	46.35 ± 18.22	58.79 ± NaN	123.87 ± 77.48	265.25 ± 154.14	132.32 ± 3.18	576.15 ± 96.75	864.79 ± 228.01	592.28 ± 499.53
	C_avg_	ng/mL	0.74 ± 0.35	1.83 ± 0.85	1.37 ± 0.51	3.86 ± 1.52	4.9 ± NaN	10.32 ± 6.46	22.1 ± 12.85	11.03 ± 0.27	48.01 ± 8.06	72.07 ± 19.00	49.36 ± 41.63
	C_max_	ng/mL	2.02 ± 0.72	4.04 ± 0.88	4.24 ± 0.78	11.89 ± 4.12	11.66 ± 7.46	26.8 ± 18.87	64.63 ± 48.38	33.93 ± 11.94	125.33 ± 14.36	128.57 ± 64.55	96.31 ± 78.85
	C_min_	ng/mL	0.42 ± 0.23	0.98 ± 0.50	0.78 ± 0.25	1.37 ± 0.58	1.17 ± 1.12	3.49 ± 2.21	6.55 ± 2.78	6.28 ± 2.74	23.03 ± 3.04	28.07 ± 4.80	15.95 ± 17.3
	Fluctuation%	%	234.38 ± 62.35	187.42 ± 67.37	263.39 ± 58.61	282.46 ± 53.13	152.68 ± NaN	244.76 ± 95.39	237.43 ± 76.46	279.10 ± 165.31	219.23 ± 63.51	185.78 ± 9.34	244.48 ± 77.38
	T_max_^a^	h	0.98 (0.97,0.98)	1.00 (0.97,1.02)	0.98 (0.97,1.00)	1.00 (0.98,1.03)	1.90 (1.90,2.00)	1.07 (1.00,2.00)	1.03 (1.02,1.95)	1.00 (1.00,2.08)	0.98 (0.98,1.00)	1.92 (1.00,4.02)	1.02 (0.95,3.95)
	R_1_	-	1.68 ± 0.19	2.01 ± 0.38	1.88 ± 0.43	1.94 ± 1.14	1.84 ± 0.20	1.81 ± 0.11	3.25 ± 1.78	1.14 ± 0.61	2.32 ± 0.50	1.65 ± 0.83	2.04 ± 0.75
	R_2_	-	2.04 ± 0.67	2.44 ± 0.84	2.76 ± 1.55	2.77 ± 2.02	5.16 ± NaN	2.96 ± 0.41	4.74 ± 2.09	2.01 ± 0.51	3.57 ± 0.39	3.51 ± 1.19	2.67 ± 0.13
	R_3_	-	/	0.93 ± 0.39	2.51 ± NaN	1.80 ± 1.46	3.76 ± NaN	1.86 ± 0.16	2.89 ± 0.99	1.22 ± 0.06	2.22 ± 0.37	2.52 ± 1.09	-
	MPratio__AUCτ_	-	0.76 ± 0.23	0.83 ± 0.24	0.40 ± 0.04	0.80 ± 0.53	0.20 ± 0.35	0.79 ± 0.30	1.00 ± 0.53	0.57 ± 0.06	1.51 ± 0.46	1.10 ± 0.38	1.26 ± 1.08
	MPratio__Cmax_	-	0.91 ± 0.10	0.61 ± 0.35	0.33 ± 0.08	0.38 ± 0.18	0.61 ± 0.64	0.52 ± 0.17	0.73 ± 0.55	0.67 ± 0.52	0.88 ± 0.33	0.84 ± 0.48	0.88 ± 0.85

a:T_max_ reported as median (range).

Blood samples were not collected after 12 h in the expansion cohort or AUC__%Extrap_>20%, so the parameters which related to elimination phase could not be calculated.

Abbreviations: AUC_0–12 h_ = Area under the concentration-time curve from 0 to 12 h after dosing, AUC_inf_, Area under the plasma concentration-time curve from the time of dosing extrapolated to infinity, AUC_τ_, Area under the concentration-time curve during a dosing interval at steady-state. AUC__%Extrap_ = Extrapolated area percentage calculated by AUC_t-inf_/AUC_inf_. C_avg_ = Average concentration, C_max_ = Maximum concentration. C_last_ = The last concentration which can be measured. C_min_ = Minimum concentration, CL/F = Apparent clearance, Fluctuation% = Percentage of concentration fluctuation, MPratio = Ratio of metabolite M8 to tunlametinib, R_1_ = Accumulation ratio calculated by C_max_ (day28)/C_max_ (PK, lead-in period or day 1), R_2_ = Accumulation ratio calculatedby AUC_τ_ (day28)/AUC_0–12 h_ (PK, lead-in period or day 1), R_3_ = Accumulation ratio calculatedby AUC_τ_ (day28)/AUC_inf_ (PK, lead-in period or day 1), T_1/2_ = Terminal half-life, T_max_ = Time taken to reach maximum concentration, V_z_/F = apparent distribution volume.

Tunlametinib was rapidly absorbed, typically attaining T_max_ within 0.50–1 h after dosing. After C_max_ was reached, concentrations of tunlametinib declined in a biphasic manner, with a t_1/2_ of 21.84–34.41 h regardless of dose level investigated. The coefficient of variation of t_1/2_ of tunlametinib was low (13.03%–47.03%). The median CL/F was within the range of 28.44–51.93 L/h. The V_z_/F was high across all dose levels (median V_z_/F was within the range of 1199.36–2009.26 L). AUC_0-t_ was higher than 80% of AUC_inf_ for each dose level except to 0.5 mg dose group. Both the average C_max_ and AUC_inf_ of tunlametinib increased with increasing dose level in an approximately dose-proportional manner in the tunlametinib 0.5–18 mg dose range with a minimum of 2.24 ng/ml and 7.03 h*ng/ml (0.5 mg) and a maximum of 349.33 ng/ml and 635.74 h*ng/ml (18 mg) for C_max_ and AUC (Table 2). In the power model analysis, the β point estimates (90% CIs) of AUC_inf_ and C_max_ after single dose of tunlametinib were 1.003 (0.870–1.136) and 1.282 (1.150–1.414), respectively (Table 3). Dose proportionality for the systemic exposure parameters of tunlametinib could not be concluded because the 90% CIs for β estimates were not completely fell within the pre-specified interval of 0.938–1.062.

**TABLE 3:** Linear evaluation of plasma pharmacokinetic parameters after single and multiple oral administration of 0.5–18 mg tunlametinib capsule in patients.

Occasion	Analyte	PK parameters	Point estimate of beta	90% CI of beta	Beta criteria
Single dose	Tunlametinib	AUC_0-12h_	1.116	(1.023,1.210)	(0.938,1.062)
		AUC_inf_	1.003	(0.870,1.136)	(0.938,1.062)
		C_max_	1.282	(1.150,1.414)	(0.938,1.062)
	M8	AUC_0-12h_	1.086	(0.923,1.249)	(0.938,1.062)
		AUC_inf_	1.148	(0.873,1.424)	(0.938,1.062)
		C_max_	1.167	(1.019,1.315)	(0.938,1.062)
Multiple dose	Tunlametinib	AUC_τ_	1.077	(0.968,1.187)	(0.938,1.062)
		C_max_	1.113	(0.936,1.289)	(0.938,1.062)
	M8	AUC_τ_	1.203	(1.023,1.383)	(0.938,1.062)
		C_max_	1.150	(0.990,1.310)	(0.938,1.062)

**FIGURE 2:**
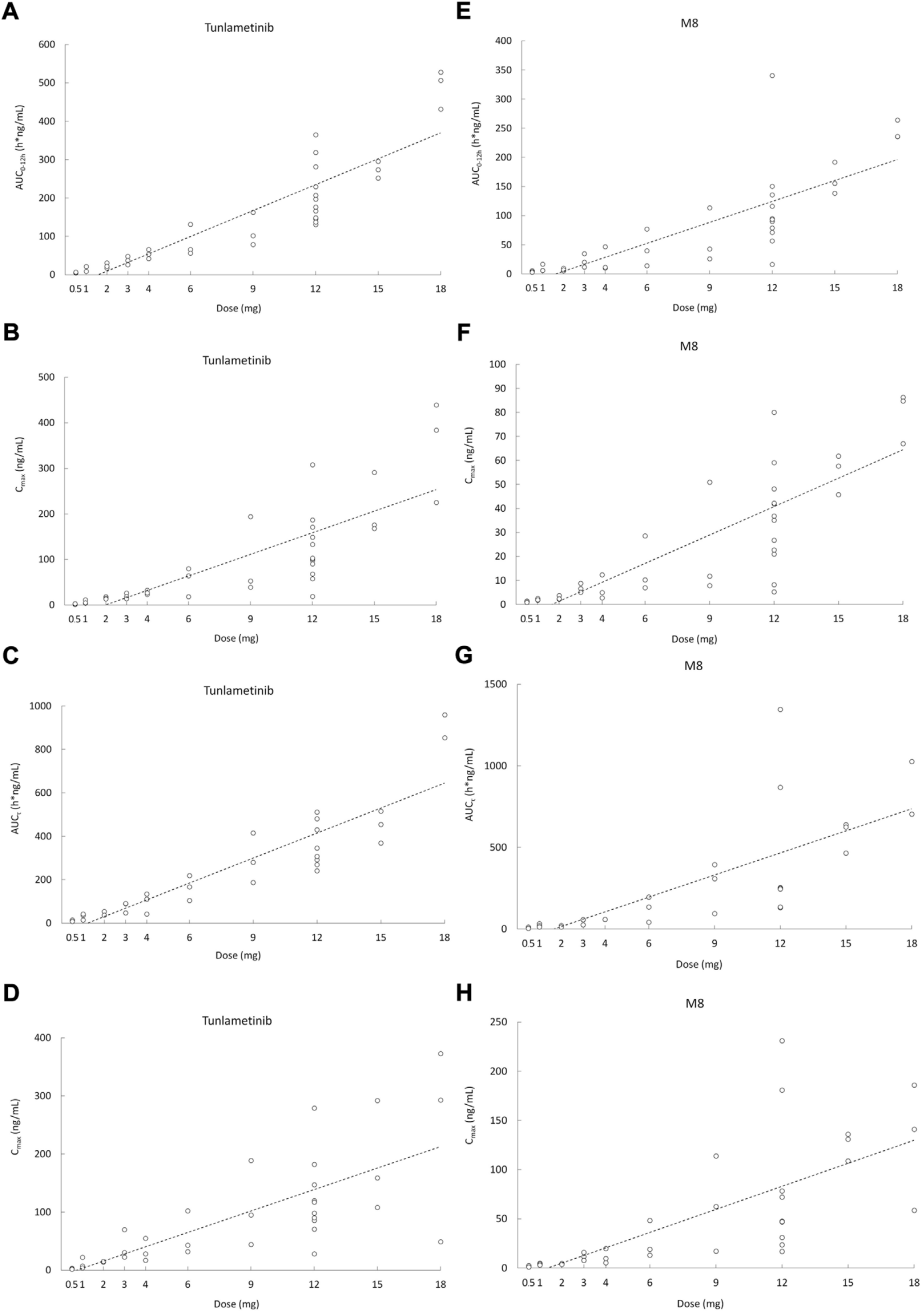
Scatter plot of AUC and C_max_ versus dose after orally administrated with 0.5–18 mg (*n* = 41) tunlametinib capsules. **(A)** single dose (AUC_0–12 h_, tunlametinib); **(B)** single dose (C_max_, tunlametinib); **(C)** multiple dose (AUC_τ_, tunlametinib); **(D)** multiple dose (C_max_, tunlametinib); **(E)** single dose (AUC_0–12 h_, M8); **(F)** single dose (C_max_, M8); **(G)** multiple dose (AUC_τ_, M8); **(H)** multiple dose (C_max_, M8).

Similarly, the main metabolite M8 was produced rapidly. Median T_max_ range of M8 was 0.97–1 h for the 0.5–18 mg twice daily tunlametinib doses. The average t_1/2_ range of M8 was 6.10–33.54 h and no dose dependency was observed for terminal half-life was detected throughout the study. The body exposure of metabolite M8 (based on AUC_inf_) was 25%–75% of that of tunlametinib (Table 2). Both C_max_ and AUC_inf_ of M8 appeared to generally increase in a dose-proportional manner in the tunlametinib 0.5–15 mg dose range (Figure 1). In the power model analysis, the β point estimates (90% CIs) of AUC_inf_ and C_max_ after single dose of tunlametinib were 1.148 (0.873–1.424) and 1.167 (1.019–1.315), respectively (Table 3; Figure 2). Dose proportionality for the systemic exposure parameters of M8 could not be concluded because the 90% CIs for β estimates were not completely fell within the pre-specified interval of 0.938–1.062.

### Multiple doses pharmacokinetics

Data from the dose-escalation phase and dose-expansion phase were pooled for multiple doses analysis according to twice daily dose level. The pharmacokinetic profiles of tunlametinib and M8 after the single dose and multiple doses of tunlametinib were similar. The concentration-time curve of tunlametinib and M8 was shown in Figure 1.

Median T_max_ range of tunlametinib was 0.50–1 h for the 0.5–18 mg twice daily tunlametinib doses. The mean accumulation ratio range of tunlametinib was 1.83–2.73, indicating minimal accumulation. Coefficient of variation of AUC_τ_ of tunlametinib was low (8.21%–47.52%) in the 0.5–18 mg range. The coefficient for variation for the C_max_ range of tunlametinib was 5.85%–70.08% (Table 2). Both C_max_ and AUC_tau_ of tunlametinib appeared to generally increase in dose-proportional manner in the tunlametinib 0.5–18 mg dose range (Table 3; Figure 1). In the power model analysis, the β point estimates (90% CIs) of AUC_τ_ and C_max_ after multiple doses of tunlametinib were 1.077 (0.968–1.187) and 1.113 (0.936–1.289), respectively (Table 3). Dose proportionality for the systemic exposure parameters of tunlametinib could not be concluded because the 90% CIs for β estimates were not completely contained within the pre-specified interval of 0.938–1.062. Dose proportionality was not proved, which might be related to the limited number of subjects.

Median T_max_ range of M8 was 0.98–1.92 h for the 0.5–18 mg twice daily tunlametinib doses. The mean accumulation ratio range of M8 was 2.04–5.16 (Table 2). Both C_max_ and AUC_tau_ of M8 appeared to increase in dose-proportional manner in the tunlametinib 0.5–18 mg dose range (Table 1; Figure 1). But in the power model analysis, dose proportionality for the systemic exposure parameters of M8 could not be concluded because the 90% CIs for β estimates were not completely contained within the pre-specified interval of 0.938–1.062 (Table 3; Figure 2).

## Discussion

Tunlametinib capsule was absorbed rapidly after administration, and the peak time of tunlametinib plasma in most subjects was within 1 h after administration. During the collection of PK blood samples in the 0.5 mg dose group, blood samples within 1 h after administration were not collected, so that the first sampling time (1 h after administration) of all subjects in this dose group was the peak time of tunlametinib and M8. Therefore, starting from the 1 mg dose group, the blood collection time points of 0.25 and 0.5 h after administration were added in each dose group. At the same time, in order to reduce the total amount of blood collection, the blood collection 3 h after administration was removed.

According to the allometric scaling model based on preclinical data, the predicted effective half-life (t_1/2_) of human is approximately 10 h. In addition, to secure the safety of patients such as dose related skin toxicity observed in pre-clinical studies, twice daily administration was selected to reduce the peak concentration of tunlametinib and minimize the potential adverse reactions during the first-in-human trial. Based on the current clinical research results, it is also proved that twice daily administration can achieve good safety and the tunlametinib exposure accumulation ration from Day 28 to Day 1 is low (1.83–2.73), supporting the BID administration. No dose-limiting toxicity (DLT) was reported during dose escalation and maximum tolerated dose (MTD) was not reached with tunlametinib doses up to 18 mg twice daily. Dose-proportional appears to increase in tunlametinib exposure. At the recommended phase II dose, the exposure profile of the tunlametinib showed low interpatient variability.

The single-dose of tunlametinib pharmacokinetics recorded rapid absorption of tunlametinib (for all doses 0.5–18 mg; median T_max_ range 0.5–1 h), which was shorter than other MEK inhibitors such as trametinib (1.0–2.08 h) (Infante et al., 2012), binimetinib (1.00–3.00 h) (Bendell et al., 2017), selumetinib (1.0–3 h) (Adjei et al., 2008) and cobimetinib (2.4–3 h) (Rosen et al., 2016). Tunlametinib could be rapidly transformed into M8 after dosing. The T_max_ of M8 was a bit longer than that of tunlametinib (≤1 h vs. 0.98–1.92 h) after multiple doses while T_max_ was similar for M8 and tunlametinib after single administration.

The decline in concentrations of tunlametinib in a biphasic manner regardless of the dose level investigated. The reason for this phenomenon may be that the absorbed tunlametinib is distributed to the tissues at a faster speed and then cleared from the body at a slower speed. The pharmacokinetic data from the multiple doses (tunlametinib in the dose range of 0.5–18 mg) were consistent with the data from the single-dose of tunlametinib. There were general dose-proportional increases in C_max_ and AUC of tunlametinib and its main metabolite M8 in the single-dose and multiple doses of tunlametinib. Rats and dogs also demonstrated proportional increase in C_max_ and AUC with increasing tunlametinib doses (unpublished data). The degree of accumulation in AUC_τ_ for tunlametinib was not obvious with one- to two-fold after multiple doses which were lower than M8 (approximately 2–5 folds). There was low interpatient variability for C_max_ and AUC of tunlametinib for most of dose cohorts including 12 mg cohort at which dose recommend phase II dose was determined (detail data will be reported elsewhere). No food effect study was performed in this study but was performed in an independent study. The pharmacokinetics profile supports twice-daily dosing of tunlametinib. In our study, the dose escalation range of tunlametinib was 0.5–18 mg, while the range was 0.125–4 mg (Infante et al., 2012) for trametinib, 10–100 mg for cobimetinib (U.S. Food and Drug Administration Center For Drug Evaluation And Research, 2015), 30–80 mg for binimetinib (Bendell et al., 2017). Consistent with the approved MEK inhibitors, tunlametinib exhibited linear PK around the therapy dose. The pharmacokinetics monitoring in each dose in the dose escalation phase contributes to the RP2D selection and the general linear PK results provide an important instruction to dose selection in clinical use. Along with the KRAS inhibitor sotorasib (Skoulidis et al., 2021), the proof-of-concept of tunlametininb as a therapeutic approach towards NRAS mutant melanoma may broaden the once challenging area of RAS mutant cancer.

In the current study, a few limitations should be noted. Firstly, blood collecting points were not designed after 12 h in the expansion cohort, therefore, we could not calculate the parameters which related to elimination phase after multiple doses of tunlametinib, such as CL/F and V_z_/F. Secondly, limited number of participants may contribute the large interpatient variability of some parameters.

## Conclusions

This phase I study showed that tunlametinib is rapidly absorbed and eliminated at a medium speed after drug withdrawal. The pharmacokinetics of tunlametinib and its metabolite suggest that twice daily dosing is appropriate for tunlametinib. The results of these phase I studies support the feasibility of further investigation of the efficacy and safety of tunlametinib in melanoma. A phase II study to assess the safety and efficacy of tunlametinib (NCT05217303) was ongoing.

## Data Availability

The original contributions presented in the study are included in the article/Supplementary Material, further inquiries can be directed to the corresponding author.
