# Supplementary material for: Phase I pharmacokinetic study of an oral, small-molecule MEK inhibitor tunlametinib in patients with advanced NRAS mutant melanoma
Source: Front Pharmacol. 2022 Nov 1;13:1039416. doi: 10.3389/fphar.2022.1039416 (PMC9663925; doi:10.3389/fphar.2022.1039416)
Supplement: Supplementary file 1 [file DataSheet1.docx]

**Supplementary Material**

**Supplementary Figure 1.**

**Part 1: Dose escalation**

**Part 2: Dose expansion**

Screening

Day -28~-7

PK run-in

Day -7~-1

Safety follow-up for 30 days

Tunlametinib 0.5~18 mg BID for 28 days

Screening

Day -28~-1

Tunlametinib at RP2D

Safety follow-up for 30 days

Single dose

Multiple doses

Multiple doses

Continued treatment

**Supplementary Figure 1: Study designs for part 1 and part 2 of this study.** PK: pharmacokinetics; BID: twice daily; RP2D: recommend phase II dose.
